# Health workforce issues and recommended practices in the implementation of Universal Health Coverage in the Philippines: a qualitative study

**DOI:** 10.1186/s12960-025-00988-3

**Published:** 2025-04-23

**Authors:** Veincent Christian F. Pepito, Arianna Maever Loreche, Ruth Shane Legaspi, Ryan Camado Guinaran, Theo Prudencio Juhani Z. Capeding, Madeline Mae Ong, Manuel M. Dayrit

**Affiliations:** 1 Health Governance Flagship Program, Center for Research and Innovation, School of Medicine and Public Health, Ateneo de Manila University, 1604 Pasig City, Philippines; 2 National Clinical Trials and Translation Center, National Institutes of Health, University of the Philippines Manila, Manila, Philippines; 3 Department of Social Sciences, University of the Philippines Manila, Manila, Philippines; 4 Provincial Government of Benguet, La Trinidad, Benguet, Philippines; 5 Benguet State University Open University, La Trinidad, Benguet, Philippines; 6 Ateneo Policy Center, School of Government, Ateneo de Manila University, Quezon City, Philippines

**Keywords:** Health workforce, Universal health coverage, Devolution, Health education, Philippines

## Abstract

**Background:**

The transition towards Universal Health Coverage (UHC) in a devolved healthcare system such as the Philippines is beset by health workforce issues considering that it is among the world’s leading source countries for health workers. This study aims to document health workforce issues and recommended practices in the implementation of UHC in the Philippines.

**Methods:**

We conducted focus group discussions and key informant interviews with health policymakers and UHC implementers in the national, regional, and local levels. Participants included local chief executives, healthcare facility administrators, and healthcare providers at tertiary, secondary, and primary levels, as well as patients. We transcribed and translated the focus group discussions and key informant interviews and analyzed it thematically.

**Results:**

Workforce factors at entry, current employment, and exit hinder the implementation of UHC. Factors at entry include: poor preparation of graduates in school for implementing UHC; difficulty in recruitment due to restrictive government hiring policies; and government budget caps for personnel services. Factors at the current employment include: poor working conditions; uncompetitive salaries; lack of trained personnel for financial management; exorbitant fees for trainings; lack of job security for nationally deployed personnel; and lack of integration of some barangay health workers and community health volunteers. Factors at exit include the pull of migration overseas and poor crisis management. Some recommended practices to recruit and retain health workforce include scholarships and return service programs; free tuition for dependents of health workers; opportunities for postgraduate degrees and specialist training overseas, and onboarding UHC training for new hires.

**Conclusions:**

To address the health workforce issues hindering the effective implementation of UHC in the Philippines, there is a need for reforms in the country’s healthcare sector and beyond. Specifically, there is a need to revisit the country’s Local Government Code, integrate further health professions education institutions and healthcare facilities, implement reforms in its basic, higher, and health education, and the civil service, revisit training costs, and training programs for specialists, and design and implement more sustainable and equitable bilateral labor agreements to keep health workforce in the Philippines and engage them as partners for optimal implementation of UHC in the country.

## Introduction

Health workers are an important building block of health systems and are vital for the implementation of health policies and programs [1]. Several studies have also demonstrated that sufficient health workers have been linked to better population health outcomes [2, 3]. For this reason, the goal of health workforce program planning is to get the right workers with the right skills in the right place doing the right things [4]. The Sustainable Development Goals Target 3.c aims to substantially increase the recruitment, development, training, and retention of health workforce in developing countries [5]. Despite this, many low- and middle-income countries around the world lack health workers in terms of absolute counts, which is further aggravated by maldistribution as they more commonly practice in more urbanized areas [6]. These shortages contribute directly and indirectly to shortages in the capacity to provide health services needed by the population. Health worker shortages prevent the attainment of Universal Health Coverage (UHC) in many countries globally [2, 7].

The Philippines has enacted its Universal Healthcare Act (UHC Act) on 2019 with the goal of providing quality healthcare at low cost [8]; however, the COVID-19 pandemic has disrupted its implementation [9–11]. Further aggravating the difficulty of implementing UHC in the country is the emigration of nurses and other healthcare professionals from the country to work overseas. The Philippines is one of the largest source countries of migrant healthcare workers, especially nurses, globally for many years [12–14]. As of 2021, the Philippines has only 7.92 physicians per 10,000 population, which is below the 10 per 10,000 ideal physician to population ratio [15]. The country is also estimated to have a shortage of 127,000 nurses, with more acute shortages in the private sector [13]. These shortages prevent the optimal implementation of health programs and prevent people from accessing healthcare services in a timely manner.

As countries transition towards UHC, it is vital to assess whether there is an adequate number of health workers with the right skills doing the right things at the right place, and whether there is adequate regulation by government and participation by private sector [4, 16]. However, while there are previous studies describing barriers to implementing Universal Health Coverage in low- and middle-income countries in general, there have been limited studies specifically focusing on health workforce issues in implementing UHC with data and perspective from both national-level policymakers to local-level implementers and patients in a devolved, health workforce source country like the Philippines [7, 13, 14, 17, 18]. Thus, this study aimed to describe and explore health workforce issues hindering UHC implementation in the Philippines and to document recommended practices for improving health workforce recruitment and retention.

## Methodology

### Study design and study population

This paper describes the health workforce issues and recommended practices as part of a larger study of the implementation issues of the UHC Act in the Philippines using the six building blocks of health framework [1]. We conducted key informant interviews (KIIs) and/or focus group discussions (FGDs) with purposively selected key health policymakers at the national and regional level, local chief executives and implementers of the UHC Act of select provinces and cities (e.g., mayors, governors, provincial/municipal/city health officers, treasurers, accountants), public and private health facility administrators and healthcare providers (e.g., physician, nurse, social workers, legal officers, medical laboratory scientists), community health workers and volunteers, and patients.

### Study setting

The Philippines is an archipelagic republic located in Southeast Asia with an estimated population of 113,000,000 and an estimated life expectancy of 72.2 years in 2021 [19]. The country has a devolved health system where the national government through the Department of Health (DOH) is responsible for setting health strategy and targets while the local governments are responsible for financing and operating local health systems, implementing health policies on the ground, and providing health services to the population. The country’s health system also has local and national government-run and taxation-funded public health facilities and a larger, market-oriented private sector. The country is administratively divided into seventeen regions, which are further subdivided into 81 provinces and 33 highly urbanized cities [20].

The local governments where most of the interviews and focus group discussions were conducted are located in a landlocked, mountainous highly urbanized city and a contiguous province independently governed from it. The province and the highly urbanized city have among the highest human development indices in the Philippines. The province’s main industries are agriculture, mining, and tourism. Meanwhile, the city is a regional center of commerce, governance, education, and culture. These local governments were selected for being implementation sites of DOH programs on UHC.

### Data collection

FGDs or KIIs were conducted either online (among national level policymakers) and face-to-face (among regional level policymakers, local chief executives and implementers, public and private health facility administrators and providers, community health workers and volunteers, and patients). The FGDs/KIIs questions for all study participants (except patients) focused on their goals for UHC implementation, their roles in UHC implementation, their approaches and strategies, the recommended practices and operational challenges they have encountered, and recommendations to improve the implementation of UHC Act in the Philippines. For patients who were all interviewed in health facilities during their visit, the questions focused on what UHC is for them and what can they recommend to improve healthcare services in their community. The study tools were pretested to primary care physicians and laypeople for clarity and ease of use. The face-to-face FGDs/KIIs were conducted in the offices and workplaces of the respondents or in their health facilities. Online FGDs or KIIs lasted anywhere between 45 min to 2 h, while face-to-face FGDs and KIIs lasted anywhere between 1 and 4 h. FGDs and KIIs were conducted between September 2023 to May 2024. All FGD/KII proceedings were audio-recorded.

### Data management, analysis, and theoretical framework

The audio recordings of the FGDs/KIIs were transcribed verbatim in English or Filipino, the languages used in the interviews. Personally-identifying information were then redacted from the transcripts. The anonymized transcripts were analyzed thematically by VCP and reviewed by the other co-authors to identify the entry, current employment, and exit health workforce issues in the implementation of UHC Act in the Philippines. Disagreements in the analysis were elevated to the senior author (MMD) for resolution.

The framework used for the thematic analysis is the Entry-Current Employment-Exit framework of the 2006 World Health Report. Briefly, entry factors are factors pertaining to education and training of human resources for health before they join the workforce, as well as recruitment. Current employment factors, on the other hand, pertain to factors that affect performance and productivity of the health workforce, including lifelong learning, salaries and working conditions. Lastly, exit factors are factors pertaining to managing migration, retirement, and attrition [4]. This theoretical framework was used both in the assessment of factors as well as the assessment of recommended practices in recruitment and retention of the health workforce.

### Reflexivity and positionality

The author team is a mix of early career and senior researchers with various levels of practical experience in the health workforce and universal health coverage. The early career researchers have conducted various qualitative and quantitative studies on the health workforce, self-care, maternal and child health, COVID-19 vaccination, nutrition, and tuberculosis. Meanwhile, the senior author (MMD) had occupied executive positions in the Philippine Department of Health and the World Health Organization, where he was Director of Human Resources for Health, and has written many papers on this issue. Four authors are physicians, two are trained in public health and epidemiology, and one in the social sciences. All researchers speak the languages used in the focus group discussions and the interviews (English and/or Filipino). All researchers are Filipinos based in the Philippines, except for one (MMO) who is based in Australia.

### Ethics

The study has received ethics clearance from the Ateneo de Manila University School of Medicine and Public Health Research Ethics Committee (AdMUREC-ASMPH_23_URC-UHC).

## Results

### Description of study participants

We conducted 17 FGDs with regional-level policymakers, local chief executives and implementers, public and private health facility administrators and healthcare providers, and community health workers and volunteers. Five (29.4%) focus group discussions were conducted with private health facilities. We also conducted 19 key informant interviews with national-level policymakers, local chief executives, public and private health facility administrators, and patients. One (5.3%) interview was conducted with the head of a private hospital network. Overall, there are a total of 92 respondents; 69 (75.0%) of respondents are females and 23 (25.0%) are males. All of the respondents are more than 18 years old. Among our respondents, 19 (20.7%) work as UHC implementers at the national, regional, and local level, 28 (30.4%) are physicians, 19 (20.7%) work as nurses, 10 (10.9%) work as barangay health workers, two (2.2%) each work as dentists, medical laboratory scientists, and information technology personnel or clerk, one (1.1%) is a lawyer, and 10 (10.9%) are patients consulting for routine childhood vaccination, hypertension, and/or other minor ailments.

### Human resources issues in the implementation of the UHC act

#### Entry factors

##### Poor preparation of graduates in school for implementing UHC

Many healthcare providers have voiced out their concerns that while their education prepared them for clinical practice, training in soft skills and government procedures and protocols were lacking. They felt that this prevented them from bringing out the best in them in implementing UHC in their communities (Quote H1, Table 1).

**Table 1:** Illustrative quotes on health workforce issues in implementing UHC in the Philippines

Quote ID	Issue	Quotation
H1	Poor preparation of graduates in school for implementing UHC	“I finished my MD from (school redacted), one of the best in the country. But when I worked here, it’s an entirely different ballgame. We weren’t trained how to deal with the local administration and procurement, how to talk to local chief executives. I wasn’t prepared nor trained for this but this is how we make things happen.” - Municipal health officer from a rural, geographically-isolated and disadvantaged area
H2	Lack of specialists and training opportunities outside Metro Manila	“If we really want Universal Healthcare, they should prioritize areas where the specialists are not concentrated like NCR. They should look at the bigger level. For example, here, we only have two cardiologists and no cardiac surgeons. So, I hope if there are TCVS (Thoracic and Cardiovascular Surgery fellowship vacancy) in Manila, if there are applicants, I wish, I hope they can prioritize applicants from other areas.” - Administrator of a public tertiary facility in an urban area
H3	Government budget caps for personnel services	“The 45% cap on personnel services really prevents us from hiring additional human resources. That’s the main reason why you see a ward nurse being assigned as the public health nurse. You see a community nurse being assigned as a records officer. That’s an extra job but there’s no extra compensation. If you notice, the health programs are not implemented properly because the program officers are working on many programs simultaneously. To be fair, I have heard that this cap is waived for UHC implementation, but I am not sure. I do not want to go to jail for it.” - Provincial level implementer from the public sector
H4	Difficulty in recruitment due to restrictive government hiring policies	“We have many job vacancies but it is hard to fill them. Government policy states that for certain positions, you can only hire those who have civil service eligibility. Not many people have that but I think there are many people who do not have civil service eligibility out there who can actually do the job. And then you have some vacancies for positions like records officers or program managers. They are looking for registered nurses for that vacancy. I mean, you do not have to be a nurse or have a PRC (Professional Regulations Commission) license to manage a health facility’s records or implement government programs. In fact, it is probably better if these people are not trained healthcare providers so that those who are trained in giving healthcare services can stick to providing care to patients. There is probably a need to revisit the eligibility requirements for vacancy positions in government.” - Provincial level implementer from the public sector
H5	Difficulty in recruitment due to restrictive government hiring policies	“The DOH has many (requirements) that are difficult to provide. Especially for manpower. We need a records officer, Information Technology administrator, but what happens is the nurse is the administrator, she is also in the records. So, the nurse is also the IT that should be designated just to meet the criteria.” - Public primary care provider from an urban area
H6	Poor working conditions	“You need an enabling environment, safe workspace, engaging them for something to make their workplace positive so that they will stay and the key there is the leader. Because if they are demotivated because of something and you ignore it, they will really leave. It’s not all about the money. I have interviewed some of them, why are you here in (office redacted)? There is more money in clinical. (They said), even if there is more money in clinical, you end up with mental health issues there because of the job, the supervisor, etc.” - Administrator of a public tertiary facility in an urban area
H7	Uncompetitive salaries in private sector relative to public sector, and public sector relative to overseas	“The problem with us here is that we will not be able to compete with (the salaries offered by) government. Especially with the salary. The entry level salary of our nurses here is just half of the entry level salary of government nurses.” - Administration of a private tertiary facility from an urban area
H8	Uncompetitive salaries in private sector relative to public sector, and public sector relative to overseas	“The nurses we lost are our best nurses. It is painful that the trained ones are the ones who leave. The ones left with us are either the new ones or are very old, because their salary (overseas) is five times what we pay here.” - Administrator of a public tertiary facility in an urban area
H9	Exorbitant fees for trainings	“For example, our medical records officer is currently in Manila undergoing training in data recording as part of DOH accreditation. And we’ve spent a lot. We’ve spent a lot. In our setting, we started May of this year (2023), as a primary care facility, we have already spent PHP 50,000 (~ USD 1,000) for trainings alone. Yes, the DOH is asking us to pay, and yet what we earned from primary care is barely PHP 2,000 (~ USD 40). These are actual data; we can show you the documents. They haven’t paid us for our previous (Konsulta) check-ups. We understand that there are processes, but realistically, how are we going to survive? Our stakeholders are questioning the wisdom of helping (in) Universal Healthcare.” - Administrator of a private primary care facility in an urban area
H10	Exorbitant fees for trainings	“And the training of the records officer, that is out of pocket, and she has to travel to Manila for that and spend PHP 20,000 (~ USD 400) just for training fees. And nobody wants to spend that much money so the renewal (of our license to operate) is still pending.” - Public primary care provider in an urban area
H11	Lack of job security for nationally deployed personnel	“Look, the nurses deployed in the NDP (Nurses Deployment Program) have been great in helping us with our health programs. However, her contract ends in a few month’s time. Thus, it will be a waste to send her to trainings considering that it is unsure if her contract will be renewed or that she will be hired again. We cannot send anybody else in the office. If only the contract of the NDPs are longer, we will not have this issue. It would also be nice because they will have benefits if they stay longer.” - Administrator of a public primary care facility in an urban area
H12	Poor crisis management	“There is a brain-drain in (agency redacted) due to recent scandals, (agency redacted) is getting pilloried (in the media and in the public), the effect to the organization is severe, and there are many policy developers before, they are gone now.” - National level implementer from the public sector
H13	Pull of migration overseas	“That’s a chronic problem. It’s (a) universal problem. Because, oh my God, medical or nursing staff, it is a problem. In our facility, we need specialists. That is why we send (our staff) abroad for fellowship because there are no offers here. And then our nurses, very big problem. Once they serve here for a year, they really go abroad.” - Administrator of a public tertiary facility in an urban area

##### Lack of specialists and training hospitals outside Metro Manila

A facility administrator lamented that the specialists and training hospitals are located in Metro Manila, leaving Filipinos in the provinces unable to access specialist care (Quote H2, Table 1).

##### Government budget caps for personnel services

A frequently mentioned barrier in implementing UHC in the Philippines is the budget cap on personnel services or salaries. This budget cap means that not more than 45% of a local government unit’s annual budget is spent on salaries of personnel. Because of this, the respondents explained that it is difficult for them to recruit adequate health workers and give them the appropriate salaries and benefits (Quote H3, Table 1).

##### Difficulty in recruitment due to restrictive government hiring policies

Another reason mentioned why there is a shortage of health workers in the public sector is the stringent requirements for applicants in job vacancies, which they deem as impractical. These requirements lead to difficulties in recruiting health workers, including those who are not patient-facing (Quote H4, Table 1). These difficulties mean that nurses who are supposed to be dedicated to providing care to patients instead spend their time doing records or information technology work (Quote H5, Table 1).

#### Current employment factors

##### Poor working conditions

A respondent from the public sector said that while the salaries in the country are relatively uncompetitive to what is offered overseas, what really drives healthcare providers away from the country are their poor working conditions here (Quote H6, Table 1).

##### Uncompetitive salaries in private sector relative to public sector, and public sector relative to overseas

Respondents from the private sector lament that their salaries for nurses cannot match the pay scale of government nurses, adversely affecting retention. As a result, they have a perennial shortage of nurses (Quote H7, Table 1). Meanwhile, respondents from government said that while the pay of nurses have increased in the past few years, it is still no match for salaries being offered overseas especially for those who are highly trained, contributing to brain drain (Quote H8, Table 1).

##### Lack of trained personnel for financial management

Respondents from both providers and health insurers revealed challenges related to the financial management capacity of healthcare facilities and local government units. Participants discussed the lack of skilled personnel to handle financial management tasks and the need for capacity building in this area. A private tertiary care facility administrator from an urban area mentioned that “usually, the common cause of fraud on the provider side is the lack of capacity among our people to handle these kinds of tasks”. They also lamented issues in the quality of trainings provided: “Instead of providing training that teaches what to do, they just have us answer things online”.

##### Exorbitant training fees

The UHC Act is a vital reform of the country’s healthcare system and with this are many new provisions and competencies needed to make it work. Because of this, there are many new training programs and capacity-building activities to train public and private healthcare providers for the new provisions and competencies needed. However, many of these trainings are not for free and some have to be paid out of pocket. Representatives from the private sector perceive the exorbitant training costs to be a disincentive towards further participating in UHC implementation (Quote H9, Table 1). This was also raised by government personnel, who were required to defray the training fees out of pocket. Because nobody is willing to pay for these trainings and their associated costs (e.g., travel and accommodation, etc.), the renewal of their facility’s license to operate is at risk of not being renewed (Quote H10, Table 1).

##### Lack of job security for nationally deployed personnel

While commending that the DOH Deployment Programs (e.g., Doctors to the Barrios, Nurse Deployment Program, Public Health Associate Deployment Programs, Post-residency Deployment Program) are vital in making UHC work in their communities, local government implementers said that it would have been better if the deployment programs are permanent because health workforce investments are wasted when the contract of the deployed personnel ends. (Quote H11, Table 1).

##### Lack of integration of some barangay health workers and community health volunteers

When we conducted focus group discussions with barangay health workers and other community health volunteers, some said that because their local government actively pursues capitation for *Konsulta* and *Konsulta + *(comprehensive outpatient primary care benefit packages), it is their job to promote these initiatives, go around the community and organize events to encourage people to register in *Konsulta*. Meanwhile, barangay health workers from other rural local governments said that they did not have any new responsibilities “since encouraging people to get COVID-19 vaccines”.

#### Exit factors

##### Poor crisis management

A national level implementer said that recent issues regarding their agency resulted in the brain drain of people who are supposed to develop policies, affecting service delivery (Quote H12, Table 1).

##### Pull of migration overseas

Another frequently mentioned issue in implementing UHC in the Philippines is a chronic, generalized shortage of healthcare workers across all cadres, mainly due to outmigration. In the words of a respondent from the public sector, it is a chronic and universal problem (Quote H13, Table 1).

### Recommended practices in recruiting and retaining health workers for UHC implementation

#### Recommended practices for entry

One of the private facilities in the study discussed that while they cannot match the salary offered by government, they do not have difficulty hiring nurses because they offer scholarship programs for nursing students who then have return service obligations. This ensures that they have sufficient nurses (Quote B1, Table 2). Another way private facilities encourage retention is by having nonmonetary benefits like scholarship programs for the children of health workers and their other dependents (Quote B2 and B3, Table 2). There are also opportunities for professional development, like free tuition fee for graduate programs (Quote B4, Table 2).

**Table 2:** Illustrative quotes on recommended practices for recruiting and retaining health workers in implementing UHC in the Philippines

Quote ID	Recommended practice	Quotation
B1	Scholarship and return service program	“Then we also have scholarship programs right now. This is primarily for nursing students. If we have, say, one student, he/she took up nursing as a scholar, because our nursing program is four years, then he/she should serve in our hospital for four years. One (year of scholarship)-is-to-one (year of return service). He/she cannot resign but he/she has a salary. We are guaranteed to have five nurses every year. This is a full scholarship. Many applicants try to avail of the said scholarship. This is our way of having, ensuring that we have enough nurses in the future.” - Private tertiary facility administrator from an urban area
B2	Free tuition for dependents	“The good thing with us is we are backed up by the university. If you are working with us, even if you have 10 children, you do not have to pay for their tuition until college. You only have to be regular. After the five probationary months once you become regular, even if you have 10 children, from grade school until college, they have free tuition.” - Private tertiary facility administrator from an urban area
B3	Free tuition for dependents	“And you, if you do not have a child, you can assign a relative (child) to avail of the educational benefit, if you are unmarried and childless.” - Private tertiary facility administrator from an urban area
B4	Free tuition for postgraduate degrees	“Also, we have professional development opportunities. If you are an employee here and you want to take your master’s (degree), that’s free.” - Private tertiary facility administrator from an urban area
B5	Specialist training overseas	“We send many of our staff for fellowship overseas because there are no opportunities here. For example, for liver cancer, our surgeons, we bring them to Korea or India. For our intensivists, who is part of the multidisciplinary transplant team, right? So, we send them to Singapore, the other one is in Thailand for diagnostic pathology. They are spread out. It’s just that we are counting the years when they will come back. And then they have a contract. They have to return.” - Administrator of a public tertiary facility in an urban area
B6	Training of personnel on UHC and embedding it in onboarding meetings	“You will see in our documentation that (as early as) 2020, we have already been talking about UHC. Now, it is difficult because they have a different understanding about it and it is difficult to talk to the clients about UHC if you yourself do not know what it is. That is what we targeted; we want to make sure that our personnel know about UHC. We have evaluation, post-evaluation to make sure that they understood. At first, only 50% had a good grasp (of UHC). We did it virtually (because of the pandemic). Then we instituted it among the new hires. When you are newly hired (in our facility), you have an orientation which includes Universal Health Coverage. It is an ongoing, continuous process so that all our employees will eventually be aware of the UHC Act. What is the UHC Act, they already know what that is.” - Administrator of a public tertiary facility in an urban area

To address the lack of opportunities for specialist training, government facilities organize scholarships for their personnel to train in other countries, in return for return service in the Philippines (Quote B5, Table 2).

#### Recommended practice for current employment

Beyond recruitment and retention, another recommended practice for UHC implementation among government facilities is training current hospital personnel about UHC and embedding UHC in onboarding training for newly hired personnel to make sure that they are aware of it and so that they can promote it among their patients and clients (Quote B6, Table 2).

## Discussion

Workforce factors at entry, current employment, and exit hinder the implementation of UHC. Factors at entry include: poor preparation of graduates in school for implementing UHC; difficulty in recruitment due to restrictive government hiring policies; and government budget caps for personnel services. Factors at current employment include: poor working conditions; uncompetitive salaries; lack of trained personnel for financial management; exorbitant fees for trainings; lack of job security for nationally deployed personnel; and lack of integration of some barangay health workers and community health volunteers. Factors at exit include the pull of migration overseas and poor crisis management. These entry, current employment, and exit factors hinder the optimal implementation of UHC in the Philippines. Some of these have been previously described in literature [4, 7, 14, 17, 21, 22], but other factors like exorbitant training fees, lack of trained personnel for financial management; the lack of engagement of community health workers, difficulty in looking for eligible candidates for vacancies, the need for health education system reform, and the temporary nature of deployment programs are some of the new barriers to UHC implementation that were identified in this study. This study also identified some recommended practices for health workers in implementing UHC in the Philippines which can be used by other facilities in the country and other low- and middle-income countries transitioning towards UHC.

One of the recommended practices to address the shortages of health workers in the Philippines are scholarships and return service agreements [23]; however, not many institutions are able to implement these properly, mainly because there is sometimes a lack of placement opportunities for graduates [24]. As an intervention, scholarships and return service agreements only work if the graduates have a guaranteed job placement once they graduate. Therefore, there is a need for greater integration between healthcare facilities and academic institutions not just to address health workforce shortages, but also to improve population health outcomes and foster economic development, as demonstrated by experiences in Brazil, Thailand, and Venezuela [25].

The implementation of the UHC will be optimized if the health workforce implementing it have the perspective and technical acumen necessary to implement it [26, 27]. Because of the curative orientation of traditional medical and health education curricula [26], primary care workers often do not have the necessary competencies for their job, such as dealing with other stakeholders in government and the private sector, conducting population health assessments, and implementing population health programs [28]. The curative orientation of traditional medical and health education curricula had also resulted in an inefficient healthcare system where patients go directly to specialists for things that are supposed to be managed at the primary care level [29]. Innovative medical curricula, both in Africa and in the Philippines, have been implemented to address this problem, but these curricula are exceptions, rather than the norm, and there have been perceptions that they produce graduates who are deficient in clinical skills [26, 30, 31]. Therefore, aligning education with the realities of UHC implementation necessitates broader changes. Some suggested reforms include a separate, specific training program for primary care [32], additional social science, public health, leadership and management courses for healthcare degree programs, and early prolonged exposure to the community [33]. Beyond primary care, there is also a need for medical specialty societies to consider equity issues in the establishment of specialty training programs and the recruitment of specialists for training. There is also a need to develop a scheme to ensure that applicants for specialist training from outside highly-urbanized areas can also get specialist training, which will help address the maldistribution of specialists in the country [34, 35]. There may also be a need for physicians in rural areas to be provided adequate avenues for professional development and career growth opportunities to encourage retention [36].

The problem goes beyond education of healthcare workers, as there is also difficulty in filling-up nonpatient facing health workforce vacancies. Respondents attribute this to the civil service eligibility requirement, which is often a requirement of many advertised government positions. However, less than 20% of civil service examinees every year pass this examination, which makes advertised positions with these eligibility requirements difficult to fill [37]. On one hand, this can be remedied by waiving these eligibility requirements as long as applicants meet other hiring criteria, but there is a more recent move to grant career service eligibility to long-time, well-performing contract of service government employees [38]. In summary, implementing UHC optimally may require general and health education and civil service reforms.

Despite allowing for a greater amount of decision space and empowerment of local governments than several countries in Africa [39, 40], the Philippines’s Local Government Code, which devolved healthcare provision to local governments [18, 41, 42], acts to adversely affect the implementation of health worker recruitment and retention practices for UHC in two ways: (a) the lack of flexibility which prevents the hiring of adequate health workforce to implement UHC; and (b) inequities in the level of implementation of UHC. According to the Local Government Code, personnel services and salaries are capped at 45% for first to third income class local governments, and 55% for lower income class local governments [40], which, in the opinion of UHC implementers, prevent them from confidently and properly fulfilling their role in implementing UHC. This cap has been waived by a more recent local budget circular to ensure that local governments absorb cost of hospital services transferred from provinces to newly created cities, salaries and benefits of health/medical personnel that may be hired to perform functions related to emergency situations, and pay salary differentials of local government-hired public health workers, among others [43]. However, there is a gray area in the circular as workers necessary to implement UHC do not necessarily fall under the provisions stipulated in the circular. This explains the reluctance of local government implementers to go beyond the budget cap and hire additional personnel for UHC implementation. Another consequence of devolution of healthcare services is the uneven implementation of UHC in the country as some local governments may choose not to prioritize health [44]. Previous studies have demonstrated that devolution has not improved health outcomes in the country and further contributed to health inequities [18, 41]. In our study, this manifested in the different level of engagements of community health workers as some local governments have implemented more UHC reforms (e.g., Konsulta and Konsulta +) than others [45, 46]. These experiences serve as a cautionary tale for other countries to study the unintended consequences of their current policies and their effects on UHC implementation. These findings also highlight the need to revisit and amend the Local Government Code to allow for flexibility in the different stages of implementing UHC and to ensure local governments implement the provisions of UHC despite devolution or through making devolution work [41, 44]. Lastly, there is a need to improve the monitoring of UHC impact measures, such as out of pocket spending and quality of care (which is closely tied to health workforce availability) among local governments to enable them to situate themselves as they transition towards universal health coverage [11, 47].

The Local Government Code also devolved the hiring of health workers to local governments [40]. However, because local governments do not or have not had the financial resources to hire sufficient numbers of health workers, the DOH instituted many deployment programs as a stop-gap solution to address these shortages [48]. These deployment programs are well appreciated by our UHC implementer respondents and in previous literature [6, 48], but these are not sustainable. Together with the Department of Interior and Local Government, the Department of Health should require local governments to come up with a long-term plan to develop and fund sufficient health workers there as a condition for receiving DOH deployed personnel, and see to it that these plans are implemented [49]. As the literature is unanimous that retention in rural and less urbanized areas is best for rural-born physicians [36, 50], this may include the establishment of local medical schools and training hospitals.

The implementing rules and regulations (IRR) of the country’s UHC Act decreed many new reforms, which necessitates capacity-building activities [51]. Although some of these capacity-building activities are made free by the DOH, there are some expensive trainings necessary for accreditation and licensing which were considered as a barrier to UHC implementation. Many study participants from both public and private facilities are reluctant to pay out of pocket for these trainings. Some participants also reported issues with the quality of these trainings. It is recommended to revisit these trainings and these training requirements, whether they can be improved, done online and/or for free or be optional; or if there can be subsidies or grants from the government and/or private sector to ensure that health workers from both public and private facilities can partake in these capacity-building activities without adversely affecting their or their facility’s financial standing.

Overseas migration of Filipino health workforce is well-documented in literature [12, 13, 21]. Although health workers have the right to migrate to other countries to improve their living standards, it results in a dearth of health workers in source countries, such as the Philippines, which leaves them with a shortage of health professionals to implement universal health coverage [52]. With the objective of protecting migrant health workers, the Philippine government has entered into bilateral labor arrangements with other countries such as United Kingdom, Spain, Norway, Japan, among other countries. However, these bilateral labor arrangements were seen as having limited effectiveness, or is unfair to the Philippines as it facilitates outmigration without regard for the sustainability of the Philippines’s health system [21, 53]. We therefore recommend that the foreign policy of health workforce source countries consider their local levels of implementation of universal health coverage as a key consideration in the design and implementation of bilateral labor agreements. This necessitates health workforce source countries to use diplomacy to create bilateral labor arrangements with destination countries that are more equitable and sustainable to ensure that they have sufficient health workforce to implement universal health coverage through capacity building, migration caps, health system investments, and technology transfer to benefit source countries as well [53, 54].

### Limitations

Our fieldwork only involved a dyad of a province and a highly urbanized city in the Philippines, which should be considered when generalizing the findings of this study. This means that health workforce factors that affect UHC implementation in conflict-prone or island areas are not documented yet, and could be pursued in future research. The study also did not touch on the issue of corruption in health workforce recruitment and retention as it was not mentioned as an issue by our respondents, even if previous studies have documented it [55, 56]. We were also unable to describe here private health workforce sector issues as described by the health labor market framework for universal health coverage, as it was beyond the scope of this article [16, 57]. Lastly, the possibility of social desirability bias cannot be discounted [58]. The short-term outcomes and long-term impacts of these recommended practices are also not formally evaluated yet. Further research may be conducted to assess the impacts of these recommended practices.

## Conclusion

The implementation of UHC in the Philippines is affected by entry, current employment, and exit factors. To help address these issues, we have also identified some best practices such as scholarships and return service programs, free tuition for dependents, free tuition for postgraduate degrees, specialist training overseas, and training of personnel on UHC and embedding it in onboarding meetings for new hires. However, to make UHC work, there is a need for reforms going beyond the health sector. Our study highlights the need to revisit the Local Government Code, integrate further health professions education institutions and healthcare facilities, and implement reforms on education, health education, and civil service sectors. There is also a need to have an equity perspective in implementing reforms, specifically, revisiting training costs so it is not discriminatory against public and private healthcare facilities and their employees who do not have the means of availing it. There is also a need to revisit the distribution of specialist training facilities and the selection of specialist physician trainees to address the maldistribution of specialists in the country. Lastly, there is a need to develop and implement bilateral labor agreements with health workforce destination countries that is more sustainable and equitable towards source countries. These reforms will hopefully help keep health workers in the Philippines to help in the implementation of UHC in the country.

## Data Availability

Anonymized data is available from the corresponding author on reasonable request.
